# Expression and Localization of a New Parvovirus-Derived Protein in the Guinea Pig

**DOI:** 10.3390/v17070893

**Published:** 2025-06-25

**Authors:** Camila E. Osega, Fernando J. Bustos, Francisca C. Bronfman, Robert J. Gifford, Gloria Arriagada

**Affiliations:** 1 Instituto de Ciencias Biomédicas, Facultad de Medicina y Facultad de Ciencias de la Vida, Universidad Andrés Bello, Santiago 8370071, Chile; 2 MRC-University of Glasgow Centre for Virus Research, University of Glasgow, Glasgow G61 1QH, UK; 3 Centre for Epidemic Response and Innovation, Stellenbosch University, Stellenbosch 7600, South Africa

**Keywords:** parvovirus, endogenous viral elements, evolution

## Abstract

Endogenous viral elements (EVEs) are genomic sequences derived from viruses. Some EVEs have open reading frames (ORFs) that can express co-opted proteins in their host. Furthermore, some EVEs that are expressed as proteins have become part of cellular genes that are fusions of hosts and EVE sequences. Endogenous parvoviral elements (EPVs) are highly represented in mammalian genomes, and some of them contain ORFs and can be expressed as proteins. We have shown that an EPV containing an ORF is part of the guinea pig gene *enRep-M9l.* This gene is broadly transcribed in vivo, indicating that it can be translated into a protein. By generating antibodies against the *enRep* coding sequence of the *enRep-M9l* ORF, we showed that the protein enRep-M9l is expressed in vivo and in the guinea pig-derived cell line JH4. By immunofluorescence and in situ proximity ligation assays, we observed that enRep-M9l protein has a cytoplasmic localization near microtubules. The results of this study suggest that the guinea pig EPV-derived protein enRep-M9l is a microtubule-associated protein. To our knowledge, this is the second demonstration that an EPV-derived protein is expressed in vivo.

## 1. Introduction

In recent years, high-throughput sequencing and new metagenomic analytical methods have revealed the widespread presence of viral-derived DNA sequences within animal genomes [1,2,3,4]. Such “endogenous viral elements” (EVEs) arise when infection of germline cells results in virus-derived DNA sequences being incorporated into chromosomes and inherited as host alleles. These EVEs constitute the viral fossil record, offering valuable insights into the evolutionary history of viruses. EVEs also reveal one of the forms viruses have impacted their host genomes: by introducing novel genetic material into the germline [5,6,7]. Although most EVE sequences found in mammalian genomes originate from retroviruses (family *Retroviridae*), an increasing number of EVEs derived from non-retroviral virus families have been reported [1,2,3,4]. Their integration is thought to occur only anomalously via non-homologous recombination (in the case of DNA viruses) or by retro-transposition of viral mRNA (in the cases of both DNA and RNA viruses) [8,9,10].

Despite the insertions, deletions, or mutations that EVEs have encountered during their evolution into their host genomes, some of them have been co-opted to perform physiologically relevant functions. In mammals, EVEs have been shown to play important roles in cell function, embryonic development, and antiviral immunity [11,12,13,14,15,16,17,18]. While most examples involve endogenous retroviruses (ERVs), co-opted EVEs derived from non-retroviral viruses that perform physiological functions have also been described [16,17,19,20,21]. For instance, human endogenous bornavirus-like nucleoprotein-2 (hsEBLN-2) encodes the mitochondrial E2 protein, which has been shown to interact with apoptosis-related proteins promoting cell survival [16]. Additionally, other EBLNs have been shown to be co-opted and expressed as non-coding RNAs (ncRNAs) that regulate gene expression [15,22]. It is now clear that sequences acquired from viruses, including those derived from parvoviruses, can be “repurposed” by animal genomes in various ways [3,4,12,15].

Parvoviruses (family *Parvoviridae*) are the most represented non-retroviral EVEs in mammal genomes [2,3,7,23,24,25,26,27,28]. Parvoviruses have highly robust, icosahedral capsids (*T* = 1) that protect their single-stranded DNA genome, which is approximately 5 kb in length. Their compact genomes are organised into two major gene cassettes: one (Rep/NS) that encodes the nonstructural replication proteins and another (Cap/VP) that encodes the structural coat proteins of the virion. All parvovirus genomes are flanked at their 3′ and 5′ ends by palindromic inverted terminal repeat (ITR) sequences; these are the only cis elements required for replication [29]. Parvovirus replication occurs in the nucleus, and occasionally viral genomes integrate into host chromosomes, facilitated by single-stranded breaks created by the nuclease function of Rep or by recombination due to homology found between the ITR regions and host genomes [30,31,32]. Therefore, their incorporation into the germline DNA and the formation of endogenous parvoviruses (EPVs) are expected to occur as a natural consequence of their biology. As with any new allele, genetic drift will rapidly eliminate most EPVs generated in such events from the gene pool. Thus, the presence of several independently acquired and fixed EPV insertions within animal genomes suggests that selective pressures have favoured their retention throughout host evolution.

Intriguingly, several EPV loci containing open reading frames (ORFs), capable of expressing complete or nearly complete proteins, have been reported [7,23,24,25,26,33,34]. Among these, we have shown that the EPV locus *EPV-Dependo.43-ODegus* is a bona fide host gene that encodes an intact Rep-derived protein, DeRep, in *Octodon degu* (degu). When ectopically expressed in cell culture, DeRep blocks parvovirus replication, strongly suggesting it has been co-opted as an antiviral gene [17,24]. We have also identified a Rep-derived EPV integrated in *Cavia porcellus* (guinea pig) as part of the cellular gene *enRep-M9l* [23]. The viral portion of *enRep-M9l* (*enRep*) was incorporated into the genome of caviomorph rodents 22–35 million years ago, while the host-derived segment originated from five exons of a *myo9*-like (*M9l*) gene. The predicted enRep-M9l protein has two isoforms due to alternative splicing, XP_013008464.2, which is 386 amino acids (aa) long, and XP_063093668.1, which is 376 aa long. Both predicted proteins contain two major domains, the N-terminal Rep_N domain and the C-terminal myosin tail domain. The Rep_N domain (aa 4-176) is homologous to the helicase domain of Rep68/78, with 34% identity to the N-terminal region of Rep68/78 of AAV2. The C-terminal myosin tail domain (aa 157-320) is homologous to the very C-terminal tail of a protein encoded in a *Myo9*-like gene of *C. porcellus*. *Myo9* genes are part of the unconventional myosin family that encode for the type IX myosins (*Myo9a* or *Myo9b*) [35]. We previously showed that (i) both isoforms of *enRep-M9l* mRNA are transcribed in guinea pig, (ii) upon cloning we were able to express a protein of the expected size in vitro, and (iii) this ectopically expressed protein localises to the cytosol [23]. Those findings suggested that *enRep-M9l* is expressed as a protein that might play a biologically relevant role in guinea pig physiology. Here, we present evidence demonstrating in vivo expression of *enRep-M9l* as a protein.

## 2. Materials and Methods

### 2.1. Cell Culture

Human embryonic kidney cells (HEK293T) were used for the production of viral particles and protein overexpression, and mouse fibroblasts cells (NIH3T3) were used to generate stable cell lines. Both cell lines were maintained at 37 °C, 5% CO_2_ in Dulbecco’s modified Eagle medium (DMEM) supplemented with 10% fetal bovine serum, 100 IU/mL penicillin, and 100 μg/mL streptomycin. *Cavia porcellus* lung fibroblasts JH4 clone 1 (ATCC CCL-158) were maintained at 37 °C, 5% CO_2_ in Ham’s F12K medium, with 10% fetal bovine serum, 100 IU/mL penicillin, and 100 μg/mL streptomycin.

### 2.2. Plasmids and Cloning

To generate pET14b-CpenRep, the coding sequence of the enRep domain of enRep-M9l was PCR amplified from pcDNA3xFLAG-enRep-M9l [23] using the primers CpenRep-NdeI-F (5′-atatcatatggtggaattttatgagctg-3′) and CpenRepstop-XhoI-R (5′-atatctcgagttacataagccctgcaccac-3′). The PCR product was digested with *Nde*I and *Xho*I, purified, and ligated into pET14b, which was digested with the same enzymes. To generate pGEX-5X3-CpenRep, pET14b-CpenRep was PCR amplified using the primers CpenRep-EcoRI-F (5′-atatggaattcaatggtggaatttatagagc-3′) and CpenRepstop-XhoI-R. The PCR product was digested with *EcoR*I and *Xho*I, purified, and ligated into pGEX-5X3, which had been digested with the same enzymes.

pMD.G encodes the Vesicular Stomatitis Virus Envelope Glycoprotein, p8.91 encodes for Gag-Pol of HIV-1, pRSVRev encodes for the Rev protein of HIV-1, and pLVX-ZgGreen1-C1 (Takara Bio USA, Inc., Mountain View, CA, USA) is a lentiviral vector that allows the expression of ZsGreen under the control of the CMV IE promoter and Puromycin resistance under the control of the PGK promoter. pLVX-ZgGreen-enRep-M9l encodes the fusion protein ZgGreen-enRep-M9l. To generate it, the enRep-M9l coding sequence was PCR amplified from pcDNA3xFLAG-enRep-M9l [23] using the primers enRepM9l-XhoI-F (5′-atatctcgagacatggtggaattttatgagc-3′) and enRepM9l-EcoRI-R (5′-atatgaattcctattcagcaggcttggcc-3′). The PCR product was digested with *Xho*I and *EcoR*I, purified, and ligated into pLVX-ZgGreen1-C1 digested with the same enzymes. pGIPZ is a lentiviral vector that allows the expression of a polycistronic mRNA containing the coding sequences of turbo GFP, puromycin resistance, and an shRNA in the mir35 context under the CMV promoter control. To generate the pGIPZ plasmids delivering the shRNA 1 and 2, two target sequences (Supplementary Figure S1) were selected using the shRNA design tool of Open Biosystems (GE Dharmacon), and oligo pairs containing the sequence were designed. The aligned oligos were cloned into *Nhe*I-digested pGIPZ by sequence independent ligation cloning.

For all the PCR amplifications, PfuUltra II Fusion High-fidelity DNA Polymerase (Agilent, Santa Clara, CA, USA) was used. All the ligations were performed using T4 DNA Ligase (New England Biolabs, Ipswich, MA, USA).

### 2.3. Generation of Stable Cell Lines

Lentiviruses were produced by co-transfection of HEK293T cells with 5 µg of pMD.G, 5 µg of p8.91, 2.5 µg of pRSVRev, and 10 µg of pLVX-ZgGreen1-C1 or pLVX-ZgGreen-enRep-M9l using polyethyleneimine (PEI) 8 mg/mL in a 3:1 proportion. Viruses were harvested 48 h after transfection, filtered at 0.45 µm, and used to transduce NIH3T3 cells in the presence of 8 µg/mL Polybrene. Cells were selected in media supplemented with 1 µg/mL puromycin.

### 2.4. Antibodies Generation

To express His-enRep, *E. coli* BL21 (DE3) were transformed with pET-14b-CpenRep. His-enRep was affinity purified from bacteria and used to immunise mice at the ImLab_USACH antibody service of Universidad de Santiago, Chile. To express GST-enRep, *E. coli* BL21 (DE3) were transformed with pGEX-5X3-CpenRep, and anti-enRep7 and 9 were then affinity purified using GST-enRep as bait.

### 2.5. Western Blot Assays

To analyse the expression of enRep-M9l in JH4 cells, 5 × 10^5^ cells were seeded in 35 mm plates. To analyse the expression of enRep-M9l untagged or fused to FLAG, HEK293T cells were transfected with 1 µg of either pcDNA3xFLAG (empty vector), pCMV-enRep-M9l, or pcDNA3xFLAG-DeRep using PEI 3:1. Forty-eight h later JH4 and HEK293T cells were lysed in Reporter lysis buffer (Promega Corporation, Madison, WI, USA). To analyse the silencing of enRep-M9l in JH4 cells, 5 × 10^5^ cells were seeded in 35 mm plates and were transfected with 1 µg of pGIPZ carrying a non-target sequence, pGIPZ-enRep-M9l shRNA1 or shRNA2. Forty-eight h later JH4 cells were lysed in Reporter lysis buffer (Promega Corporation, Madison, WI, USA). Samples were then boiled in 5× sodium dodecyl sulphate (SDS) loading buffer, and the proteins were resolved by 10% acrylamide SDS-PAGE. After transfer to PVDF membranes, the blots were probed with mouse serum 7, mouse serum 9, mouse anti-Flag Clone M2 (Sigma, Saint Louis, MO, USA), and mouse anti-α-tubulin Clone DM1A (Sigma, Saint Louis, MO, USA) all in 1:1000 dilution or rabbit anti-pan Actin (D18C11) mAB (Cell Signaling, Danvers, MA, USA) 1:5000. Secondary antibodies conjugated to HRP and the ECL reagents were used for developing. To analyse the expression of enRep-M9l in the guinea pig, tissues were obtained from a fresh male *C. porcellus*. All experiments were performed according to the protocol approved by the Bioethical Committee of Universidad Andres Bello (Acta 002/2018). Upon euthanasia, testis, lungs, liver, kidney, spleen, heart, and brain were isolated and rinsed with ice-cold phosphate saline buffer (PBS) and cut into small pieces, and protein extracts were prepared using RIPA buffer (50 mM Tris pH 8.0, 150 mM NaCl, 0.1% SDS, 1% Triton X-100, 0.5% sodium deoxycholate) with protease inhibitors. Samples were homogenised with 20 Dounce strokes and maintained for 30 min at 4 °C with rotation. Finally, samples were centrifuged for 20 min at 10,000× *g* at 4 °C. Supernatants were quantified, and 30 µg of protein was used for western blot assays, using the affinity-purified mouse anti-enRep7 (1:500), anti-enRep9 (1:500), or mouse anti-GAPDH (1:1000).

### 2.6. Immunofluorescence

NIH3T3 cells expressing ZsGreen or ZgGreen-enRep-M9l cells were seeded on glass coverslips. After 24 h, cells were fixed with 4% paraformaldehyde (PFA) for 20 min at room temperature, washed with PBS-Ca^2+^/Mg^2+^ (137 mM NaCl, 2.7 mM KCl, 10 mM Na_2_HPO_4_, 1.8 mM KH_2_PO_4_, CaCl_2_ 180 μM, MgCl_2_ 1 mM; pH 7.4), and permeabilised with 0.5% Triton X-100 in PBS for 5 min. Cells were then blocked with 1% bovine serum albumin (BSA) in PBS (PBS-BSA) for 1 h at 37 °C in a humid chamber. Coverslips were incubated overnight at 4 °C with anti-enRep7 or anti-enRep9 1:50 in PBS-BSA. After washing, cells were incubated for 1 h at room temperature with anti-mouse IgG-AlexaFluor 594 (Thermo Fisher A21207, Waltham, MA, USA) 1:500 in PBS-BSA. JH4 cells were fixed and permeabilised as above and incubated with anti-enRep7 or anti-enRep9 1:50 and anti-mouse IgG-AlexaFluor 488 (Thermo Fisher A11001, Waltham, MA, USA) 1:500. After washing, nuclei were stained with Hoechst 33342 (1 μg/mL) for 15 min. Coverslips were mounted with Fluoromount-G (EMS, Hatfield, PA, USA).

JH4 cells were also co-incubated with anti-enRep9 1:50 and phalloidin-AlexaFluor 594 (Thermo Fisher A12381, Waltham, MA, USA) 1:500; anti-enRep9 (1:50) and rabbit anti-α-actin (Bioss bs-0189R, Woburn, MA, USA) 1:50; or anti-enRep9 and rabbit anti-β-tubulin (Abcam ab6046, Cambridge, MA, USA) 1:100. Anti-mouse IgG-AlexaFluor 488 and anti-rabbit IgG-AlexaFluor 555 (Thermo Fisher A21429, Waltham, MA, USA) were used at a dilution of 1:500. Nuclei were stained, and cells were mounted as above.

All images were acquired using sequential laser excitation on a Nikon C2+ confocal microscope (Nikon, Melville, NY, USA) equipped with a Nikon plan Apo100x/NA1.40 oil immersion objective (Nikon, Melville, NY, USA). Imaging processing was performed using FijI (version 2.16) [36].

### 2.7. Proximity Ligation Assay

The in situ proximity ligation assay (PLA) was performed using the Duolink In Situ Red Starter Kit (Sigma-Aldrich, Saint Louis, MO, USA), according to the manufacturer’s instructions. JH4 cells were seeded on glass coverslips, fixed with 4% PFA for 20 min at room temperature, and permeabilised with 0.5% Triton X-100 in PBS for 5 min. After washing, cells were blocked with Duolink blocking solution for 1 h at 37 °C in a humidified chamber and incubated with primary antibodies diluted in Duolink antibody diluent overnight at 4 °C. The following combinations were used: mouse anti-Histone H3 (ab9050 Abcam, Cambridge, MA, USA) 1:250 and rabbit anti-β-tubulin 1:100; mouse anti-α-tubulin 1:50 and rabbit anti-β-tubulin 1:100; mouse anti-enRep9 1:25 and rabbit anti-α-actin 1:12; or mouse anti-enRep9 1:25 and rabbit anti-β-tubulin 1:100. After washing with buffer A, cells were incubated with anti-mouse PLUS and anti-rabbit MINUS PLA probes for 1 h at 37 °C, followed by ligation for 30 min at 37 °C. Amplification was carried out for 100 min at 37 °C in the dark. Then, coverslips were incubated with anti-rabbit IgG-AlexaFluor 488 (Thermo Fisher A11034, Waltham, MA, USA) 1:500, anti-mouse IgG AlexaFluor 488 1:500, anti-rabbit IgG-AlexaFluor 647 (Thermo Fisher A31573, Waltham, MA, USA) 1:500, or anti-mouse IgG-AlexaFluor 647 (Thermo Fisher A31571, Waltham, MA, USA) 1:500 in Duolink antibody diluent for 1 h. Finally, cells were washed twice with Buffer B and once with 0.1× Buffer B, and they were mounted with ProLong antifade mounting medium (Thermo Fisher P36930, Waltham, MA, USA). Images were captured and processed as described for immunofluorescence.

## 3. Results

### 3.1. The EPV-Derived Protein enRep-M9l Is Expressed in Guinea Pig Cells and Tissues

**Figure 1:**
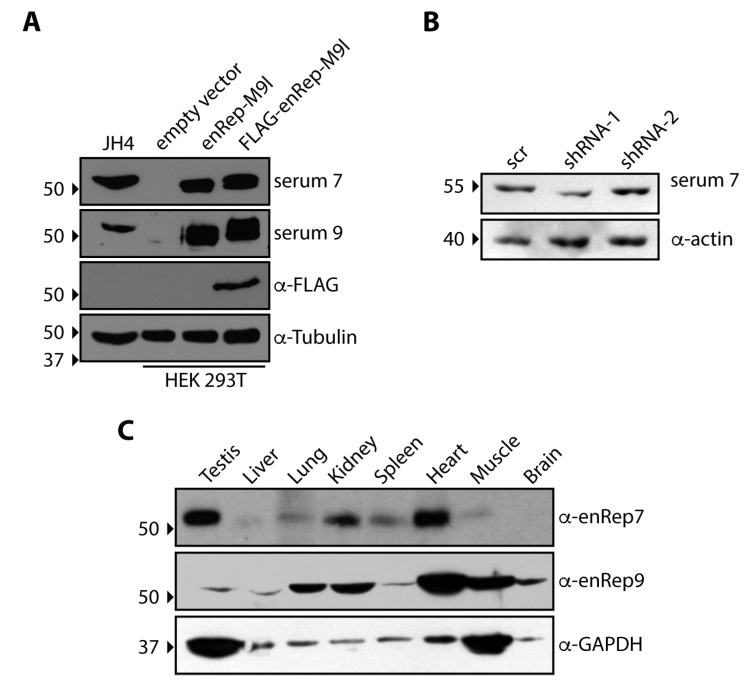
The EPV-derived protein enRep-M9l is expressed in guinea pig cells and tissues. (**A**) HEK 293T cells were transfected with plasmids encoding enRep-M9l, FLAG-enRep-M9l, or with empty vectors. Forty-eight h post-transfection, cells were lysed, and western blots were performed using serum 7, serum 9, or anti-FLAG. A lysate of the guinea pig-derived cell line JH4 was included in the western blot. Tubulin was used as a loading control. A representative experiment of at least 3 independent assays is shown. (**B**) JH4 cells were transfected with plasmids encoding a scrambled shRNA or shRNAs directed against the enRep-M9l mRNA. Forty-eight h post-transfection, cells were lysed, and western blots were performed using serum 7 or anti-actin. (**C**) Different organs of the guinea pig were isolated, lysed, and analysed by western blot using the affinity-purified anti-enRep7 or anti-enRep9. Samples from one representative animal of the two analysed are shown. GAPDH was used as a loading control. The migration of the molecular weight marker is indicated on the left-hand side of each blot. The antibodies used in each western blot are indicated on the right-hand side.

We have previously ectopically expressed in vitro a protein derived partly from an EPV encoded in the gene *enRep-M9l* of *Cavia porcellus* (guinea pigs) [23]. Since this gene is widely transcribed in guinea pig tissues [23], we asked if it is also expressed as a protein. For this purpose, we immunised mice with the enRep domain of enRep-M9l and obtained several sera that were subsequently tested by western blot. To this end, we used cell lysates from HEK293T cells transfected with plasmids encoding untagged enRep-M9l, a FLAG-tagged version, or an empty vector as control. Additionally, lysates from JH4 cells, a cell line derived from lung fibroblasts of guinea pig, were included. We observed that in HEK293T the serums only detected a band at the expected size (just above the 50 kDa) when the cells were transfected with the plasmids encoding enRep-M9l. Moreover, when FLAG-enRep-M9l was transfected, a higher molecular weight band was detected, as expected by the tag addition (Figure 1A). The anti-FLAG antibody only detected a band when cells were transfected with the plasmids encoding FLAG-enRep-M9l (Figure 1A, third panel). In JH4 cells, both serums could recognise a band at the expected size, indicating that enRep-M9l protein is expressed in the guinea pig cell line (Figure 1A, first column). To confirm that the band observed in JH4 cells corresponds to enRep-M9l, JH4 cells were transiently transfected with plasmids delivering two distinct shRNAs targeting the coding sequence in the mRNA (Figure S1). We observed that only shRNA1 was able to reduce the intensity of the band after 48 h of transfections (Figure 1B). The blotting of the same membrane against the loading control (actin) shows that more protein was loaded in the shRNA1 line, indicating successful silencing of the protein. These results confirm that enRep-M9l protein is expressed in the guinea pig cell line.

To investigate the protein expression of enRep-M9l across various guinea pig tissues, we performed a western blot analysis on protein lysates from the testis, liver, lung, kidney, spleen, heart, muscle, and brain. We affinity-purified the anti enRep antibodies from the serums 7 and 9, designated α-enRep7 and α-enRep9. As shown in Figure 1C, both antibodies detected a protein band at the expected molecular weight of approximately 50 kDa, consistent with the predicted size of enRep-M9l. However, the two antibodies displayed markedly different tissue-specific detection patterns. The α-enRep7 antibody revealed strong signals in the testis, spleen, and heart, with weaker bands in the liver, lung, and kidney. Notably, this antibody failed to detect the protein in muscle or brain lysates. In contrast, the α-enRep9 antibody detected the protein in all tissues examined. The strongest signals with α-enRep9 were observed in the heart and muscle, with moderate expression in the lung, kidney, and brain. The detection of the protein in the brain by α-enRep9 is a key finding, demonstrating its presence in the central nervous system. Overall, these results confirm that the enRep-M9l protein is widely expressed, and this protein-level evidence is consistent with our previous findings of broad transcriptional activity [23]. The differential detection between the two antibodies may reflect differences in their affinity or the accessibility of their respective epitopes in various tissues.

**Figure 2:**
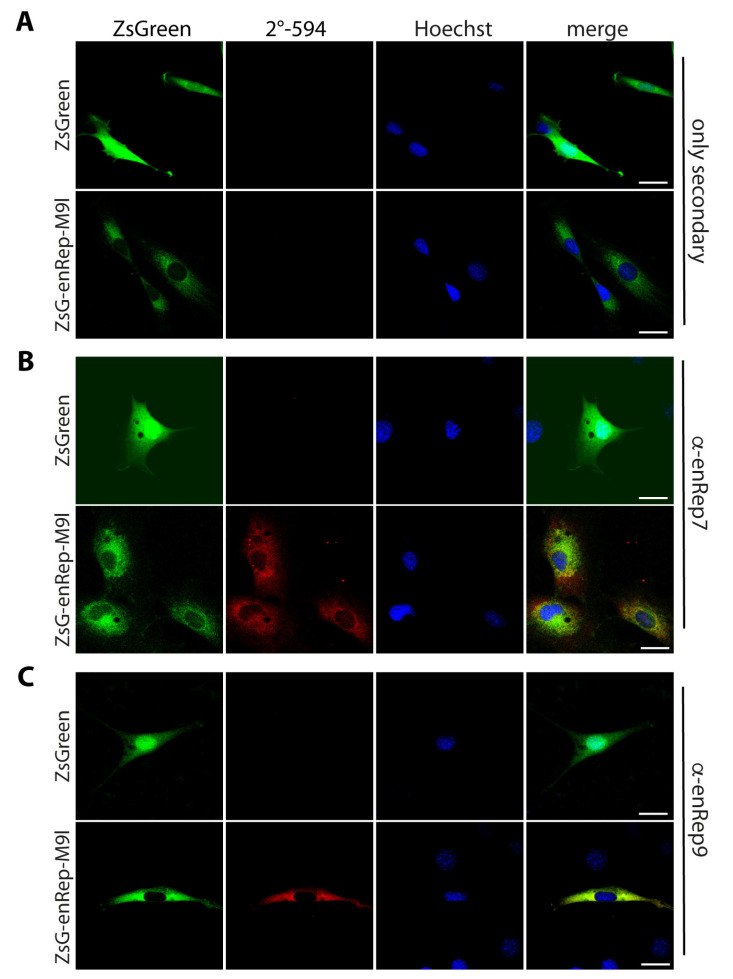
Antibodies raised against enRep recognise the translational fusion ZsGreen-enRepM9l in situ. NIH3T3 cells expressing ZsGreen or ZsGreen-enRepM9l were seeded in coverslips, fixed and stained with anti-mouse IgG-AlexaFluor 594 (**A**), mouse anti-enRep7 (**B**), or mouse anti-enRep9 (**C**) followed by anti-mouse IgG-AlexaFluor 594 and nuclear staining with Hoechst 33342. The summatory of all confocal planes is shown for each condition. Scale bar = 20 μm.

To test if the antibodies work in immunofluorescence, NIH3T3 cells expressing ZsGreen or ZsGreen-enRep-M9l were used. While ZsGreen is in the cytoplasm and the nucleus, ZsGreen-enRep-M9l signal is observed mainly in the cytoplasm as expected, due to the absence of nuclear localization signal in the amino acid sequence (Figure 2A). Next, we used the purified antibodies to conduct immunofluorescence of enRepM9l. In ZsGreen-expressing cells, neither antibody showed a signal (Figure 2B,C upper panel). In ZsGreen-enRep-M9l-expressing cells, both antibodies showed a signal that in some areas overlapped with the green fluorescence (Figure 2B,C bottom panel), indicating that the affinity-purified antibodies can be used to detect enRepM9l in situ specifically.

**Figure 3:**
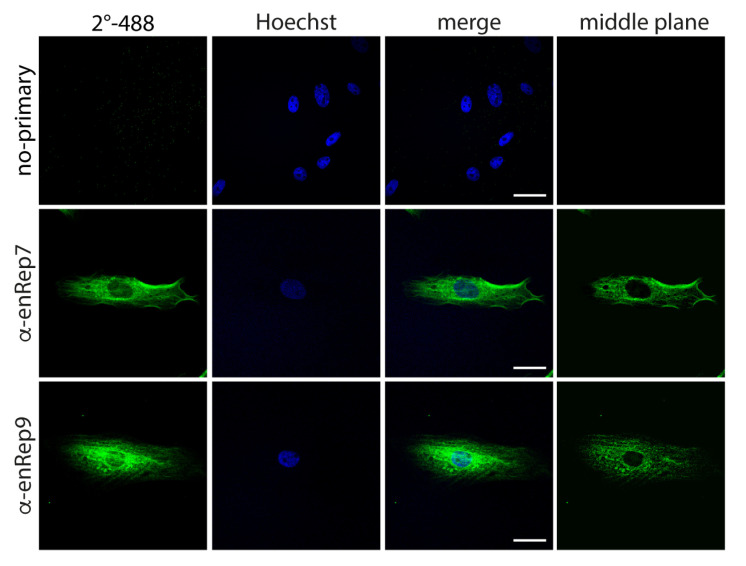
enRep-M9l localises in the cytoplasm of guinea pig cells. JH4 cells were seeded in coverslips, fixed, and stained with anti-mouse IgG-AlexaFluor 488 (**upper panel**), mouse anti-enRep7 (**middle panel**), or mouse anti-enRep9 (**bottom panel**) followed by anti-mouse IgG-AlexaFluor 488 and nuclear staining with Hoechst 33342. The summatory of all confocal planes is shown in the first three columns for each condition. The confocal middle plane on the green channel is shown on the right-hand side column. Scale bar = 20 μm.

Since we detected enRep-M9l by western blot in JH4 cells (Figure 1A,C), and the anti-enRep antibodies detected specifically ZsGreen-enRepM9l by immunofluorescence in NIH3T3, we attempted to detect the endogenous protein in JH4 cells by immunofluorescence. We observed a clear cytoplasmic signal with both antibodies, and no signal when only the secondary antibody was used (Figure 3). Interestingly, both antibodies reveal a partially filamentous signal excluded from the nucleus when the middle confocal plane is observed. Since a similar pattern was observed with anti-enRep9 in overexpressed and endogenous enRep-M9l, we decided to perform all further experiments with this antibody (anti-enRep from now on). Our western blot and immunofluorescence results indicate that enRep-M9l is a cytoplasmic protein expressed in the guinea pig.

### 3.2. The Protein enRep-M9l Is in Close Proximity to Microtubules

The protein enRepM9l is expressed in vivo, and the signal observed with both antibodies in the immunofluorescence assay in JH4 revealed a pattern that resembles microfilaments and microtubule cell distribution. This agrees with the fact that enRep-M9l is derived in part from the last exons of a *Myo9*-like gene, and it is predicted to have a myosin tail domain. Thus, we analyse whether enRep-M9l co-distributes with microfilament or microtubules in JH4 cells.

**Figure 4:**
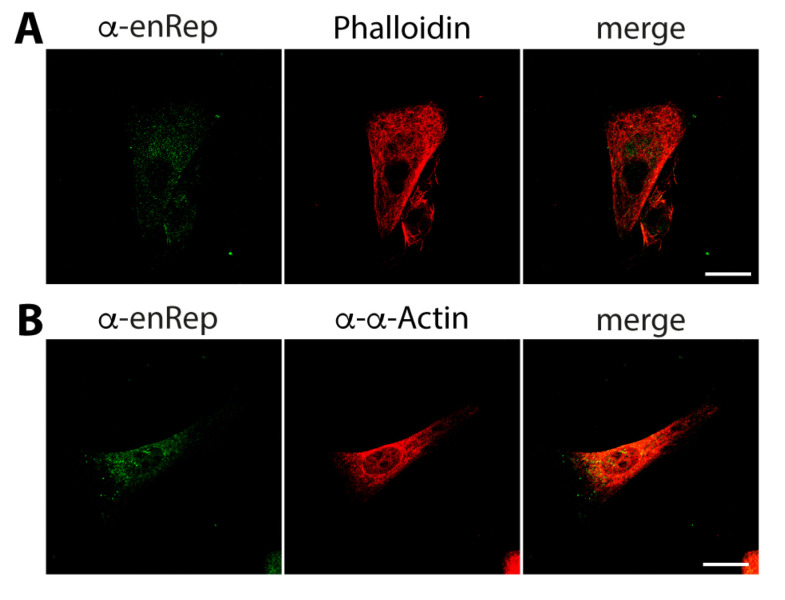
enRep-M9l does not co-distribute with microfilaments. JH4 cells were seeded in coverslips, fixed, and stained with anti-enRep and phalloidin-AlexaFluor 594 (**A**) or mouse anti-enRep and rabbit anti-α-actin (**B**), followed by anti-mouse IgG-AlexaFluor 488 (**A**,**B**) or anti-rabbit IgG-AlexaFluor 555 (**B**). The confocal middle plane of a representative cell for each assay is shown. Scale bar = 20 μm.

First, we asked if enRep-M9l co-distributes with actin filaments. We co-stained JH4 cells with anti-enRep and Phalloidin-AlexaFluor 594 to test this. We observed the cytoplasmic distribution of both signals, and there was no clear overlap between them (Figure 4A). We also performed co-staining using a rabbit anti-α-actin antibody. Importantly, this was the only anti-actin antibody that gave us a signal in immunofluorescence assays in guinea pig cells. This antibody detects both the globular and filamentous forms of actin; thus, the signal is filamentous and randomly distributed throughout the cell. We found no clear overlap or co-distribution of the actin signal with enRep-M9l (Figure 4B).

**Figure 5:**
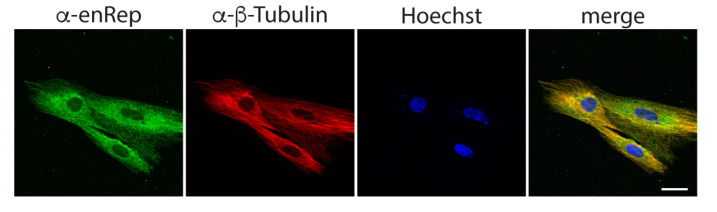
enRep-M9l co-distributes with microtubules. JH4 cells were seeded in coverslips, fixed, and stained with mouse anti-enRep and rabbit anti-β-tubulin followed by anti-mouse IgG-AlexaFluor 488, anti-rabbit IgG-AlexaFluor 555, and nuclear staining with Hoechst 33342. The confocal middle plane of a representative group of cells is shown. Scale bar = 20 μm.

Next, we asked if enRep-M9l co-distributes with microtubules. For this, we performed immunofluorescent assay using anti-enRep and anti-β-tubulin antibodies. We observed a cytoplasmic distribution for enRep-M9l that appears partially filamentous and a clear filamentous distribution with nuclear exclusion signal for β-tubulin. More importantly the signals overlap, as shown in the yellow signal in the merged panel of a single confocal plane (Figure 5). This suggests that enRep-M9l associates with or is in close proximity to microtubules, possibly interacting with them.

**Figure 6:**
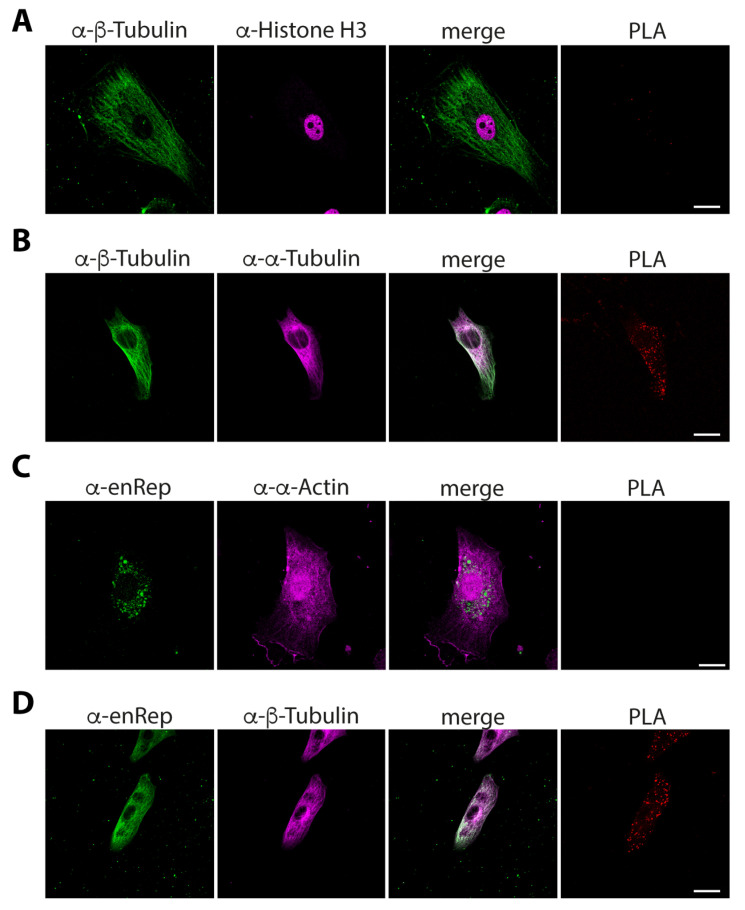
enRep-M9l is in close proximity to microtubules in guinea pig cells. JH4 cells were seeded in coverslips, fixed, and PLA assays for different targets were performed. (**A**) JH4 cells were stained with rabbit anti-β-tubulin and mouse anti-histone H3. (**B**) JH4 cells were stained with rabbit anti-β-tubulin and mouse anti-α-tubulin. (**C**) JH4 cells stained with mouse anti-enRep and rabbit anti-α-actin. (**D**) JH4 cells were stained with mouse anti-enRep and rabbit anti-β-tubulin. (**A**,**B**) After PLA reaction, the cells were incubated with anti-rabbit IgG-AlexaFluor 488 and anti-mouse IgG-AlexaFluor 647. (**C**,**D**) After PLA reaction, the cells were incubated with anti-mouse IgG-AlexaFluor 488 and anti-rabbit IgG-AlexaFluor 647. The confocal middle plane of a representative cell for each assay is shown. Scale bar = 20 μm.

To determine if enRep-M9l is close to actin or microtubules we performed proximity ligation assays (PLAs). First, we tested how PLA-negative and -positive assays look in JH4 cells. We co-stained the cells with rabbit anti-β-tubulin and mouse anti-histone H3 for the negative control and performed the PLA reaction. We observed the cytoplasmic localization of tubulin and the nuclear localization of histone H3, with no overlapping in the merged image and hence no signal for the PLA assay (Figure 6A). We co-stained the cells with rabbit anti-β-tubulin and mouse anti-α-tubulin as a positive control. We observed the cytoplasmic localization of tubulin with both antibodies, a clear overlap as a white signal in the merged image and red puncta on the PLA assay, indicating a positive signal showing close proximity between the targets (Figure 6B). Next, to test the proximity to actin filaments, PLA was conducted using mouse anti-enRep and rabbit anti-α-actin antibody. We found no PLA signal using this combination (Figure 6C). Finally, we co-stained the cells with anti-enRep and rabbit anti-β-tubulin and observed the cytoplasmic localization of both proteins with overlap indicated by a white signal in the merged image. More importantly, we observed a spread of red puncta on the PLA assay (Figure 6D), indicating that enRep-M9l is in close proximity to tubulin. Together, our data demonstrate that enRep-M9l protein is expressed in vivo and suggest that it is a microtubule-associated protein.

## 4. Discussion

Intact EPVs have been found in a surprisingly high frequency in mammalian genomes, suggesting that their conservation is associated with a potential physiological role in their host [1,3,23,24]. We and others have identified EPVs derived from *Rep* genes of dependoparvoviruses that contain intact ORFs and are transcribed in their hosts [23,24,33]. Here, we provided evidence of the in vivo expression of enRep-M9l. Aligned with our previous report showing broad transcription of *enRep-M9l* [23], the protein was found in every analysed tissue, with all the antibodies tested, and in the guinea pig-derived JH4 cell line. Unlike DeRep, a protein derived from an almost intact EPV with nuclear localization [17,23], enRep-M9l is a chimeric protein, partially derived from an EPV and partially from the host. The EPV that was incorporated into the genome of caviomorph 22–35 million years ago has lost most of its sequence, including the Rep nuclear localization signal. Thus, the new chimeric protein is located in the cytoplasm and co-distributes with microtubules.

Our previous work has shown that *enRep-M9l* mRNA exists in two isoforms that are spliced differently between exons 5 and 6, with the shorter isoform lacking 10 codons and maintaining the reading frame [23]. The long and short isoforms encode proteins of 386 or 376 amino acids, respectively. Since the difference between the two isoforms is only 10 amino acids, our polyclonal antibodies were unable to differentiate between them by size. As some tissues, like the spleen, express both mRNA isoforms [23], determining if both proteins are simultaneously or differentially expressed between tissues of the guinea pig or at different cell-cycle stages will required more fine-tuned tools, such as monoclonal antibodies that can differentiate between the isoforms. The isoforms may have unrelated functions in the cell, which will be a matter for future studies.

Although we were able to obtain tissues from guinea pig to perform the western blot assays, there is a limitation in performing genetic analysis to understand if this new protein is essential during development or in specific tissues of this animal. It would be interesting to generate more antibodies that can detect the protein in situ, allowing for histological analysis of where in the different tissues and organs of guinea pig the protein is expressed. Fortunately, cell lines derived from guinea pig are available. The guinea pig-derived cell line JH4 allowed us to study the localization of the endogenous enRep-M9l protein in situ. We demonstrated a co-distribution with microtubules but not with actin filaments. This was also observed when FLAG-enRep-M9l was overexpressed in JH4 cells. The PLA assays revealed that enRep-M9l is located less than 40 nm away from tubulin, suggesting a potential role as a microtubule-associated protein and opening a new avenue for studying the function of enRep-M9l using guinea pig cell lines.

Although we have not yet determine the exact function of enRep-M9l, we can speculate that the predicted helicase domain of the Rep/NS segment of enRep-M9l may act as a polymerization domain, as replicases form multimeric structures necessary for parvoviral replication [37,38,39]. These putative multimers could decorate the microtubules, explaining the filamentous distribution of both ZsGreen-enRep-M9l and endogenous enRep-M9l. This pattern has been observed in microtubule regulators that form multimers, such as some members of the TRIM family [40,41]. The C-terminal (aa 157-320) of enRep-M9l is homologous to the very C-terminal tail of a protein encoded in a *Myo9*-like gene of *C. porcellus*. *Myo9* genes are part of the unconventional myosin family that encode for the type IX myosins (*Myo9a* or *Myo9b*) [35]. These unconventional myosins mediate cytoskeleton dynamics through a Rho GTPase-activating domain (RhoGap) located at the C-terminal tail [35,42,43]. This region is where the homology between *enRep-M9l* and the *Myo9*-like resides. Rho GTPases, such as RhoA, are molecular switches that regulate cytoskeletal dynamics; they alternate between an active (GTP-bound) and an inactive (GDP-bound) state, and this process is controlled by regulatory proteins such as guanine exchange factors (GEFs), guanine dissociation inhibitors (GDIs), and GTPase-activating proteins (GAPs) [44]. Although RhoA is classically associated with regulating actin and stress-fibre formation, its involvement in regulating microtubule organization and stability has also been described. This influence is exerted primarily through the activation of effectors such as Rho-associated kinase (ROCK), which can phosphorylate microtubule-associated proteins [45]. It was also reported that RhoA could be involved in the capture of microtubule plus ends, as well as in their spatial orientation during processes such as cell migration or division [46]. RhoGAP activity, like that of unconventional type IX myosins, allows for RhoA inactivation by stimulating GTP hydrolysis. In this scenario, enRep-M9l could participate in the modulation of these pathways through its putative RhoGap domain. Although RhoGAP activity has not been demonstrated in this protein, its cytoplasmic localization and close association with microtubules suggest that enRep-M9l influences the microtubule network, either through local regulation of RhoA or through structural interactions with microtubule-associated proteins.

Our finding mirrors observations from hsEBLN2, where the fusion of an EVE and endogenous cellular sequences gave rise to a new gene with a distinct cellular function [16]. Thus, our work has increased the evidence that viral genes have been repurposed as new functional genes encoding proteins in animal genomes. To our knowledge, this is the second demonstration that an EPV-derived protein is expressed in vivo.

## 5. Conclusions

The guinea pig gene *enRep-M9l*, which is partially derived from an EPV, is expressed as a protein in vivo. The cytoplasmic localization of the enRep-M9l protein, along with its close proximity to microtubules, indicate that it functions as a microtubule-associated protein.

## Data Availability

All data supporting this work are available upon request.

## Supplementary Materials

The following supporting information can be downloaded at: https://www.mdpi.com/article/10.3390/v17070893/s1, Figure S1: The mRNA sequence of enRep-M9l is shown. The AUG for translation intitiation and UAG stop codon are depicted in capital bold red letters. The target sequence of the shRNA 1 and 2 are underlined the nucleotide that border the enRep and M9l regions is depicted in capital bold black.
