# Supplementary material for: Expression and Localization of a New Parvovirus-Derived Protein in the Guinea Pig
Source: Viruses. 2025 Jun 25;17(7):893. doi: 10.3390/v17070893 (PMC12300724; doi:10.3390/v17070893)
Supplement: Supplementary file 1 [file viruses-17-00893-s001.zip › viruses-3696543-supplementary.pdf]

1 guguuauuccaguguagcaacuuggcagcacaaaccugaguugucauucaggggaaacugaaagcuacag
71 agcggcaggugacucaugccaucaucacgccugaaccaagagcggagaugguauaaaagggcugggagag
141 cgaggaucaguucucuucaugcagagagacaucaac**AUG**guggaauuuaugagcugauuguucacauucc
211 cagugauguggagcaacaccuuccagcaacugacucagaucccaucagcgaauggagccuaccagaggac
281 agcgacuugguuuugagugaauuugagaaaucucaacugaccaucgccaauaaaauucauaaggaaauuc
351 uagggaaaugggaaaaaaauaacccaaaagucuuuaggcaguuuaaaaaaggaacaaaaucuuuugagcu
421 ucgcauuuugauugaaacuucuauaaugauccccaccucucugggucagaguuugaggcaauuuaaauuu
561 acaugccuaaaucauguguaucccggcauucuacaguggauguaugccuccaaaaaaccugauggauccu
631 augagacuccuggugaggauuacaucaacaaaucccuucugacagagagccaagcagaguugcaguuguu
701 guggaccaacauggaugaguacaaagagccagcucaaaauuucucaggacgcaagaggcugguagaggag
771 caucagaaccaaagucugucagaucugucccaagcaagc**G**cuucuggcaguuguggugcagggcuuaugg
841 cccagauaaaggacggccuccauaagcuggaagacauacaagcuuucggggaggaaauccugacacaggc
911 cauaagaaugaaaagaagcugaagaacacggaggcugagaugauucagcugcaggaacuggaagcagcug
981 agcgugccaagugccaggcccagcaggagcgggacgagcugacugaggagccugccaacagcagcggcaa
1051 aagagcccuagaggggaaaccucagcuggagacccgcacuucccagcuggaccugaucaaugaccggcug
1121 aagagggucagccugcagauugaugagaucaccacuaugaucuccacauggaaggccuccaucacugacu
1191 cugagaccaagggaacaaagagggaggagcagcuggauaucgagaccaaguacgugccccaugguuuccc
1261 acuucacaggcacgaggacaugaagcuugucuugguacaagaagugucacagaugggcacuggugacugc
1331 ucagaugaggaggaagaugcaaagaugauggggccgaggccaagccugcugaa**UAG**gcugcuucuccugu
1401 ucucaggaaggacagauggaugacugcccaccucucccugccccugcagcacccugccuugggacaacug
1471 ugaccugauccuuacccuaguccugcugcccugcugagggcauuggcuuccucuggugcagcccuccagc
1541 ccuucccccucccagaaucugacaccaaagagucagggcugggcccaggccugcacagugugaccagcag
1611 gccugcacccucugcugucugaaaccguggugaggcaaggagggcaggcugcuggggcgggcuccagggu
1681 uuucuacaaaucuauuuuuauucagacuaaaagaguuuugauagccucacacccagaaguguugcagccc
1751 gcacagcuccagcccccaccuggccaccagcuccuccuuccugcugccuugcugcuggcaaucacacaug
1821 gugaccucacaccucuguccugugggcccccacuccccugggcccugagugguucugaaaggagcaggccc
1891 agcuccaccucugugcaggacacagaaugcaagggagggggacuucuuauccccucugggcagcccucu
1961 gucacugcccuccugauuccaagaugucacaaauguaguaccacugccccuccugcacuggucuaucugg
2031 uuggugauuuauuuguauucguaauugacuuguauaugcuucauuagacucacuucuggcaagaagacuu
2101 uuuuuauaaaggaaacaaacuugcaucaaauuaugaagcuacauauaauacaaagcucugaguucagguc
2171 ccaguugcugucacaaaggagugaguggaacuccgacccaccccuuuuucuauaaugaaauugccuuagc
2241 augaguugcagcugcaccaccucaguaagcugguuuacagauguuuucacugagucacaauaaagagcac
2311 cauuugcugagaa

Supplementary figure 1. The mRNA sequence of enRep-M9l is shown. The AUG for translation intitiation and UAG stop codon are depicted in capital bold red letters. The target sequence of the shRNA 1 and 2 are underlined the nucleotide that border the enRep and M9l regions is depicted in capital bold black .

Metodología

Se utilizaron dos pares de oligonucleótidos diseñados para el silenciamiento específico de la transcripción de enRepM9l: pGIPZ3 (5'-TTCGCATTTTGATTGAAAC-3'), y pGIPZ4 (5'-TCAGGACGCAAGAGGCTGG-3'). Ambos fueron diseñados considerando regiones específicas dentro de la porción viral del transcrito (enRep), con el fin de evitar homología con el gen endógeno de miosina IX-like (*Myo9-like*) de *Cavia porcellus*, y así minimizar el riesgo de efectos off-target. Las secuencias fueron seleccionadas utilizando la herramienta de diseño de shRNA de Open Biosystems/GE Dharmacon), que permite identificar sitios óptimos para silenciamiento, priorizando regiones con alta accesibilidad y especificidad para mejorar la eficiencia del knockdown y reducir posibles interferencias inespecíficas.

Los oligonucleótidos diseñados para la secuencia blanco fueron resuspendidos en TE pH 8.0 a una concentración final de 100 pmol/µl. Para obtener una solución de 10 µM (10 pmol/µl), se preparó una mezcla de alineamiento con 1 µl del oligo forward, 1 µl del oligo reverse, 2 µl de Buffer 2 NEB (10×; 50 mM NaCl, 10 mM Tris-HCl, 10 mM $MgCl_2$, 1 mM DTT, pH 7.9) y 16 µl de H_2O libre de nucleasas. La mezcla fue incubada a 95 °C durante 3 minutos y luego se dejó enfriar lentamente a temperatura ambiente para permitir la hibridación.

El vector pGIPZ fue previamente linealizado mediante digestión con NheI por 3 horas a 37 °C y luego digerido con una T4 DNA polimerasa (NEB, Cat. NEB.M0203S) con actividad exonucleasa 3´→5´ en presencia de Buffer 2 de NEB por 2 minutos a 37 °C. El producto fue corrido en gel de agarosa a 20 V por una noche y el fragmento lineal purificado utilizando el kit NucleoSpin® Gel and PCR Clean-up (Macherey-Nagel, Cat. Nº 740609.50).

La ligación de los oligos alineados al vector linealizado se realizó mediante la enzima T4 DNA ligasa (NEB, Cat. NEB.M0202S), siguiendo las condiciones recomendadas por el fabricante. Posteriormente, se transformaron células competentes de *E. coli* DH5α y se sembraron en placas con antibiótico. Como control negativo se utilizó un vector pGIPZ-scramble (scr).

Las colonias seleccionadas fueron corroboradas mediante digestión con las enzimas de restricción NheI y NotI, la cual permite identificar clones positivos por la pérdida del sitio de restricción NheI. Finalmente, los plásmidos clonados fueron enviados a secuenciación para confirmar la inserción correcta del shRNA.
